# A Crop Canopy Localization Method Based on Ultrasonic Ranging and Iterative Self-Organizing Data Analysis Technique Algorithm

**DOI:** 10.3390/s20030818

**Published:** 2020-02-03

**Authors:** Fang Li, Xiaohu Bai, Yongkui Li

**Affiliations:** 1 College of Engineering, Shenyang Agricultural University, Shenyang 110866, China; happy-lifang@163.com; 2 School of Mining Engineering, Liaoning Shihua University, Fushun 113001, China

**Keywords:** canopy localization, fuzzy clustering, self-organizing algorithm, ultrasonic sensor, sprayer boom height control

## Abstract

To protect crops from diseases and increase yields, chemical agents are applied by boom sprayers. To achieve the optimal effect, the boom and the crop canopy should be kept at an appropriate distance. So, it is crucial to be able to distinguish the crop canopy from other plant leaves. Based on ultrasonic ranging, this paper adopts the fuzzy iterative self-organizing data analysis technique algorithm to identify the canopy location. According to the structural characteristics of the crop canopy, based on fuzzy clustering, the algorithm can dynamically adjust the number and center of clusters so as to get the optimal results. Therefore, the distances from the sensor to the canopy or the ground can be accurately acquired, and the influence of lower leaves on the measurement results can be alleviated. Potted corn plants from the 3-leaf stage to the 6-leaf stage were tested on an experiment bench. The results showed that the calculated distances from the sensor to the canopy using this method had good correlation with the manually measured distances. The maximum error of calculated values appeared at the 3-leaf stage. With the growth of plants, the error of calculated values decreased. The increased sensor moving speeds led to increased error due to the reduced data points. From the 3-leaf stage to the 5-leaf stage, the distances from the sensor to the ground can also be obtained at the same time. The method proposed in this paper provides a practical resolution to localize the canopy for adjusting the height of sprayer boom.

## 1. Introduction

Nowadays, boom sprayers have been commonly used for the application of pesticides, herbicides, and fertilizers in agriculture. It is known that an optimal boom height is essential for achieving an even spray distribution [1,2,3]. In order to achieve a reasonably uniform application of such materials, it is necessary that the boom be maintained at a constant distance from the target area (crop canopy or ground). The performance of the boom height control system is partially dependent on the accuracy of the plant height measurement [4]. To control the boom height, ultrasonic sensors are mounted to the sprayer boom for measuring the distance of the boom either from the canopy or the ground [5,6,7,8].

Ultrasonic sensors have been widely used in past decades for agricultural applications because of their low price, simple information processing, and mature technology. Ultrasonic sound is transmitted and then reflected by the measured object. The distance can be calculated based on the sound speed and the travel time between sound transmission and echo return. Ultrasonic sensors for the determination of plant heights have been used for weed detection. The results showed that weed presence was correctly predicted in more than 92% of the cases [9]. Thomas et al. [10] concluded that the prediction accuracy of forage mass from ultrasonic height measurements is acceptable. Shrestha et al. [11] investigated ultrasonic sensing approach as a kind of technology for vehicle-based corn height estimation. They found a good correlation between the measured and estimated crop plant height using an ultrasonic sensor. Kataoka et al. [12] attached an ultrasonic sensor to the front head of a sprayer that was driven along the crop row to measure the vertical distance between the sensor and plant canopy. They concluded that the ultrasonic sensor preformed better for soybean and corn height measurements than a laser beam sensor. Chang et al. [13] developed an ultrasonic plant height measurement using three ultrasonic sensors and incorporated it into a commercial mechanical harvester to measure wild blueberry plant height. Liu et al. [14] designed a precise application system for the sprayer that used an ultrasonic sensor to collect the distance from the boom to the canopy and the ground. The system mainly used the significant difference between the ground height and the crop canopy height to determine whether there is a crop under the nozzle, thereby achieving the purpose of precise spraying. Wei et al. [15] designed an online control system of spray boom height and balance, using ultrasonic sensors to measure the distance from the boom to the ground. In order to eliminate the influence of the leaf occlusion on the ground distance detection, a spray boom height signal filtering algorithm for removing the branches and leaves interference was adopted. Wang et al. [16] designed a boom height automatic adjusting system that used ultrasonic sensors to measure the distance between the boom and the ground to prevent the boom from colliding with the ground.

During the spraying process, the ultrasonic sensor mounted on the boom goes forward along with the sprayer to scan the plants. As a result, the data acquired by the sensor may include the distance from the sensor to different levels of leaves and the ground. An effective method is needed to separate these signals from the sensor. Otherwise, the distance value to the canopy extracted from the data would not be very accurate. However, there have not been any reports about how to avoid the influence of leaves beneath the canopy on the determination of the canopy location under the sensor. In this paper, a method was proposed to distinguish the canopy from the rest of leaves and the ground. In addition, experiments were conducted to evaluate the influence of the plant growth stage and moving speed of the sensor on the precision of the proposed method.

## 2. Materials and Methods

### 2.1. Experimental Apparatus

The experimental apparatus consists of a motion control system and a data acquisition system.

**Figure 1:**
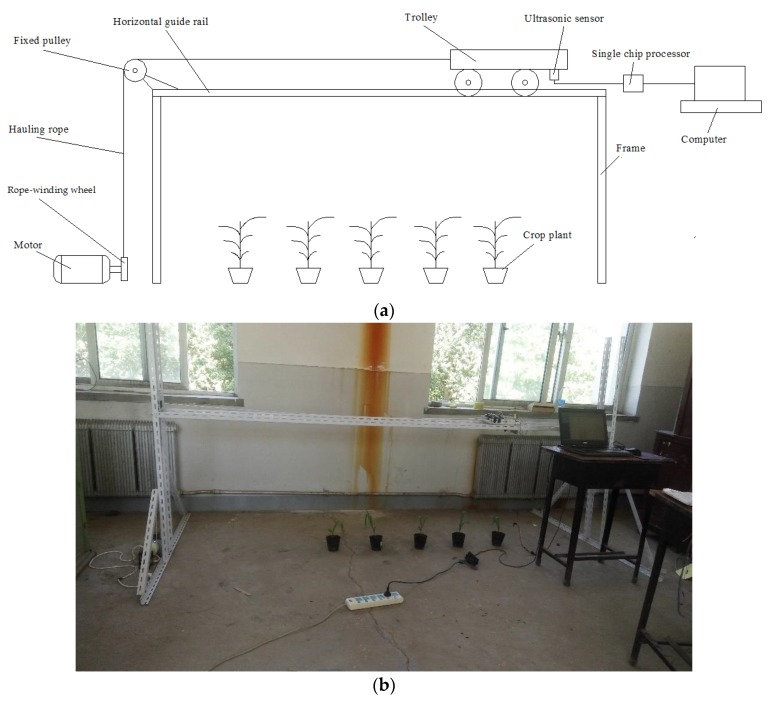
Experimental apparatus: (**a**) Schematic diagram; (**b**) Actual photo.

As shown in Figure 1, a horizontal guide rail was mounted on the top of the frame to allow a trolley to move along it. Crop plants were placed below the rail at an interval of 0.3 m. An ultrasonic sensor pointing straight down to the plants was fixed to the trolley. The distance from the ultrasonic sensor to the ground was 0.81 m. A fixed pulley was placed on one end of the rail. An electrical motor was used to pull the trolley through a hauling rope. One end of the hauling rope was fixed to the rope-winding wheel, which was installed on the motor shaft, while the other end was mounted to the trolley via the fixed pulley. As the trolley moved, the ultrasonic sensor on the trolley can scan the crop plants under it to measure the distance from the sensor to the plants. The motor is a speed adjustable one (80YT25DV22, Jingyan Automation Component Co. Ltd., Xiamen, China) that can provide a rotation speed of 900–1400 r/min with the support of a SF25E controller. During the experiment, the speed of the sensor was in the range of 0.5–6 km/h, which is comparable to the actual operation speed of the sprayer in the field.

Our choice of the ultrasonic sensor KS109 (manufactured by Guide Electromechanical Technology Co. Ltd., Shenzhen, China) comes with noise reduction functionality. The transducer ultrasonic wave frequency is 40 kHz with the beam angel of 10°. The sensing range is 0.08–11 m with the allowing temperature of 0–60 °C. A temperature sensor (DS18B20) is embedded in the ultrasonic sensor to compensate for the effect of temperature fluctuation on the electronics. A single chip processor (STC12C5A60S2, Hongjing Technology Co. Ltd., Shenzhen, China) was used to control the transmission and receiving of the ultrasonic wave and calculate the travel time. Then, the travel time was sent to a computer to conclude the corresponding distance, which was stored for further analysis. The canopy height can be acquired by subtracting the distance between the sensor and the canopy from the distance between the sensor and the ground. For every plant, its canopy height was also measured with a measuring tape manually for comparisons. At the 3-leaf, 4-leaf, 5-leaf, and 6-leaf growth stages respectively, five corn plants were randomly chosen and tested.

### 2.2. Data Processing Method

Since the data acquired by the ultrasonic sensor include the distance to the crop canopy, the distance to the underlying leaves, the distance to the soil, and so on, the data in different classes are far from each other. Fuzzy clustering analysis can establish a fuzzy similarity to classify data based on the characteristics and similarity [17,18]. Iterative Self-Organizing Data Analysis Technique Algorithm (ISODATA) can dynamically modify the clustering center and get local optimal results according to the sample characteristics. Furthermore, it can dynamically adjust the number of clusters based on the local data to obtain the best clustering results [19,20,21]. Therefore, this paper proposed an algorithm of fuzzy ISODATA that used ISODATA to identify parameters based on fuzzy clustering. 

#### 2.2.1. Basic Principle

First, the data samples are divided into several clusters. Then, the fuzzy matrix is modified repeatedly according to the objective function until the matrix has converged. 

The set of samples to be clustered is $X=\left\{X_{1},X_{2},\cdots ,X_{n}\right\}$. If each sample has *d* characteristic indicators, i.e., $X_{i}=\left(x_{i1},x_{i2},\cdots ,x_{id}\right)$, then the characteristic indicator matrix is

(1) $$X=\left[\begin{array}{c} X_{1}\\ \begin{array}{c} X_{2}\\ \vdots \\ X_{n} \end{array} \end{array}\right]=\left[\begin{array}{cccc} x_{11} & x_{12} & \cdots & x_{1d}\\ x_{21} & x_{22} & \cdots & x_{2d}\\ \vdots & \vdots & \ddots & \vdots \\ x_{n1} & x_{n2} & \cdots & x_{nd} \end{array}\right].$$

Define *c* cluster center vectors as

(2) $$V=\left[\begin{array}{c} V_{1}\\ \begin{array}{c} V_{2}\\ \vdots \\ V_{c} \end{array} \end{array}\right]=\left[\begin{array}{cccc} v_{11} & v_{12} & \cdots & v_{1d}\\ v_{21} & v_{22} & \cdots & v_{2d}\\ \vdots & \vdots & \ddots & \vdots \\ v_{c1} & v_{c2} & \cdots & v_{cd} \end{array}\right].$$

Divide the sample *X* into *c* clusters (2 ≤ *c* ≤ *n*), then the fuzzy clustering matrix is

(3) $$U_{c\times n}=\left[\begin{array}{c} U_{1}\\ U_{2}\\ \vdots \\ U_{c} \end{array}\right]=\left[\begin{array}{cccc} u_{11} & u_{12} & \cdots & u_{1n}\\ u_{21} & u_{22} & \cdots & u_{2n}\\ \vdots & \vdots & \ddots & \vdots \\ u_{c1} & u_{c2} & \cdots & u_{cn} \end{array}\right]$$

where *u_ij_* is the membership degree of the *j* th sample to the *i*th cluster, which satisfies the following conditions: (1) $u_{ij}\in \left[0,1\right],\forall i,j$; (2) $\sum_{i=1}^{c}u_{ij}=1,\forall j$; (3) $0<\sum_{j=1}^{n}u_{ij}<n,\forall i$. 

In order to obtain the optimal clustering, the following objective function should be minimized.

(4) $$J\left(U,V\right)={\sum_{i=1}^{c}\sum_{j=1}^{n}\left(u_{ij}\right)}^{q}{\left\|X_{j}-V_{i}\right\|}^{2}$$

where ${\left\|X_{j}-V_{i}\right\|}^{2}$ is the distance between the *j* th sample and the *i* th cluster center, and *q* is the weighted index. Bezdek has proved that *q =* 2 is the optimal by experiments [22]. 

Construct a Lagrange function:

(5) $$L\left(U,V,\lambda \right)=\sum_{i=1}^{c}\sum_{j=1}^{n}{\left(u_{ij}\right)}^{q}{\left\|X_{j}-V_{i}\right\|}^{2}+\sum_{i=1}^{n}\lambda_{i}\left(\sum_{i=1}^{c}u_{ij}-1\right)$$

where *λ* is Lagrange multiplier.

Taking the partial derivative of Equation (5) with respect to *u_ij_* and *V_i_*, and then setting it to zero, we obtain the expression of the membership degree and the cluster center when Equation (5) takes a minimum value. 

The expression of membership degree is:

(6) $$u_{ij}=\frac{1}{{\sum_{p=1}^{c}\left[\frac{{\left\|X_{j}-V_{i}\right\|}^{2}}{{\left\|X_{j}-V_{p}\right\|}^{2}}\right]}^{\frac{1}{q-1}}}$$

where *X_j_* is the *j* th sample, *V_i_* is the *i*th cluster center, and *V_p_* is the *p*th cluster center.

The expression of the cluster center is

(7) $$V_{i}=\frac{\sum_{j=1}^{n}{\left(u_{ij}\right)}^{q}X_{j}}{\sum_{j=1}^{n}{\left(u_{ij}\right)}^{q}}.$$

#### 2.2.2. Split and Merge

The two main factors affecting the spray quality are the distances from the boom to the crop canopy and to the ground. In our analysis, these two distances were extracted from the two clusters whose centers are the closest and the farthest from the boom, respectively. In order to achieve more accurate distance measures, the members of the two clusters were further adjusted with merge and split operation based on ISODATA.

(1) Split

Calculate the average distance from each sample in a cluster to the cluster center $\bar{D_{i}}$ using the following formula:

(8) $$\bar{D_{i}}=\frac{1}{n_{i}}\sum_{X\in \omega_{i}}\left\|X-V_{i}\right\|,i=1,2,\cdots ,c$$

where *n_i_* is the number of samples in the *i* th cluster, *X* is the sample in the *i* th cluster, *V_i_* is the *i* th cluster center, and *ω_i_* represents the *i* th cluster.

Calculate the average distance $\bar{D}$ from every sample in sample set *X* to the cluster center in which the sample is located using the following formula:

(9) $$\bar{D}=\frac{1}{n}\sum_{i=1}^{c}n_{i}\bar{D_{i}}$$

where *n* is the number of samples in sample set *X*.

Calculate the standard deviation *σ_i_* of the the *i* th cluster *ω_i_* using the following formula:

(10) $$\sigma_{i}=\sqrt{\frac{1}{n_{i}}{\sum_{X\in \omega_{i}}\left\|X-V_{i}\right\|}^{2}}.$$

If $\sigma_{i}>\theta_{S}$, $\bar{D_{i}}>\bar{D}$, and $n_{i}>2\left(\theta_{n}+1\right)$ where *θ_S_* is a preset standard deviation value, *θ_n_* is the minimum number of samples in a cluster that can be set according to the sprayer speed; then, *ω_i_* can be split into two clusters of which the cluster center are the followings:

(11) $$\begin{array}{l} V_{i}{}^{\left(2\right)}=V_{i}-0.5\sigma_{i}\\ V_{i}{}^{\left(1\right)}=V_{i}+0.5\sigma_{i}. \end{array}$$

(2) Merge

Calculate the distance *D_ij_* between two different cluster centers. If $D_{ij}<\theta_{c}$ where *θ_c_* is the minimum distance between two cluster centers, then the two clusters are merged into a new cluster. Its center is as follows:

(12) $$V_{k}=\frac{1}{n_{i}+n_{j}}\left(n_{i}V_{i}+n_{j}V_{j}\right)$$

where *V_k_* is the center of the new cluster, *n_i_* and *n_j_* are the numbers of samples in the *i* th and *j* th cluster, and *V_i_* and *V_j_* are the centers of the *i* th and *j* th cluster.

#### 2.2.3. Algorithm Procedures

(1) Set the cluster number *c* (2 ≤ *c* ≤ *n*), the initial fuzzy clustering matrix *U*^(0)^, the number of iterations *k* (*k* = 0), and the threshold value *ε* (*ε* > 0).

(2) Calculate the cluster center of the *k* th iteration using Formula (7):

(13) $$V^{\left(k\right)}={\left[V_{1}{}^{\left(k\right)},V_{2}{}^{\left(k\right)},\cdots ,V_{c}{}^{\left(k\right)}\right]}^{T}.$$

(3) Modify the fuzzy clustering matrix *U*^(*k*)^ of the *k* th iteration applying Formula (6).

(4) Compare *U*^(*k*)^ with *U*^(*k*+1)^. If $\max\left\{\left\|u_{ij}^{\left(k+1\right)}-u_{ij}^{\left(k\right)}\right\|\right\}\leq \varepsilon$, then stop the iteration, and the current clusters are optimal. Otherwise, set *k* = *k* + 1 and go back to Step (2).

(5) Split or merge the clusters according to Formulas (11) and (12).

(6) Make the clusters distinct. For $\forall X_{j}\in X$, if $\left\|X_{j}-V_{k}\right\|=\underset{1\leq i\leq c}{\min}\left\|X_{j}-V_{i}\right\|$, then the sample *X_j_* is classified into the *k* th cluster.

#### 2.2.4. Clustering Effect Test

Evaluate the effect of the fuzzy ISODATA method using the following formula:

(14) $$v_{PE}\left(U\right)=-\frac{1}{n}\sum_{j=1}^{n}\sum_{i=1}^{c}u_{ij}\ln\left(u_{ij}\right)$$

where *v_PE_*(*U*) is the average fuzzy entropy of the fuzzy clustering matrix.

The smaller the value of *v_PE_*(*U*), the less the uncertainty in clustering and the better the clustering effect.

## 3. Results and Discussion

### 3.1. Algorithm Verification

**Figure 2:**
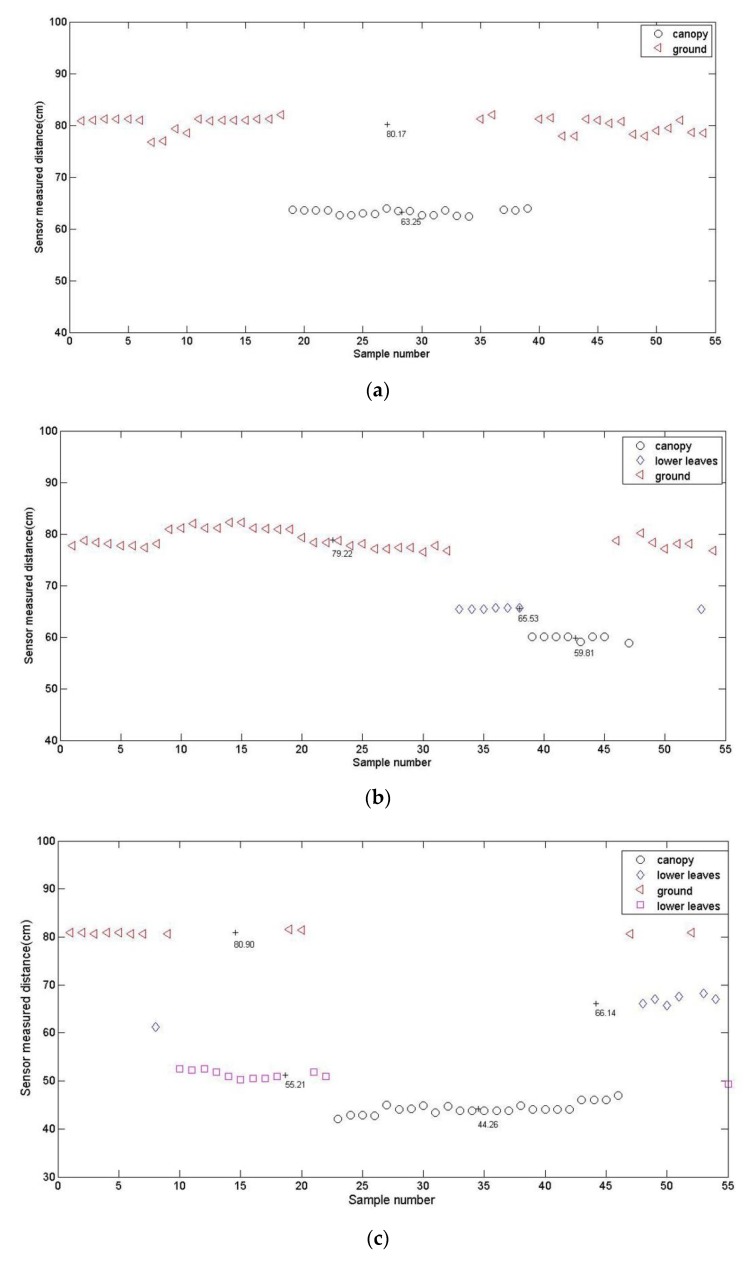

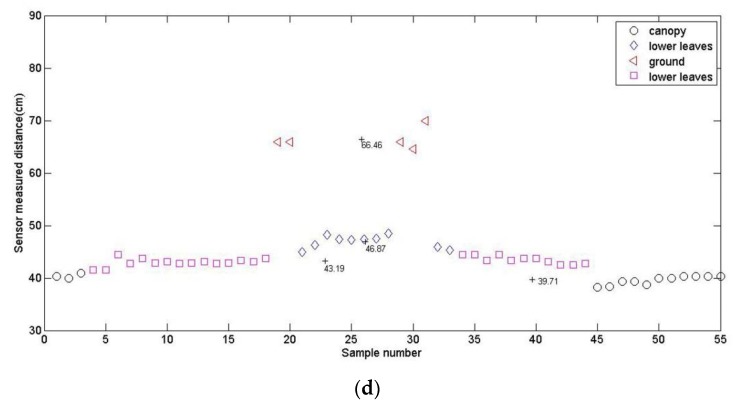
Clustering results for corn plants at different growth stages: (**a**) 3-leaf stage; (**b**) 4-leaf stage; (**c**) 5-leaf stage; and (**d**) 6-leaf stage.

At the beginning, the data measured by an ultrasonic sensor was divided into 3 clusters that represented the crop canopy, the lower leaves, and the ground, respectively. Therefore, the initial number of clusters *c* is 3. To ensure the reliability of the clustering results, the threshold value *ε* is generally between 10^−4^ and 10^−6^. So, in this paper, it was set to 0.0001 in order to reduce the number of iterations. As for the minimum number of samples *θ_n_*, the standard deviation *θ_S_* and the minimum distance between two cluster centers *θ_c_*, we performed tests by setting different values and verified the accuracy of the clustering results with some known sample data sets. Then, we selected the values that provided the best clustering results as the preset values. Finally, they were set to 20, 5, and 2, separately. The corn plants from the 3-leaf stage to the 6-leaf stage were scanned by an ultrasonic sensor and the data were clustered with the proposed algorithm. The results are shown in Table 1 and in Figure 2.

For corn plants of the 3-leaf stage, the cluster center value 63.25 and 80.17 represented the distances from the sensor to the canopy and the ground, respectively. For corn plants at the 4-leaf stage, the cluster center values 59.81, 65.53, and 79.22 were the distances from the sensor to the canopy, to the lower leaves, and to the ground. For corn plants at the 5-leaf stage, there were 4 clusters. Except for the distances from the sensor to the canopy and the ground, the distance from the sensor to the lower leaves was classified into 2 clusters. Their centers were 51.21 and 66.14. For corn plants at the 6-leaf stage, there were also 4 clusters. Nevertheless, the maximum value of cluster center 66.46 can not represent the distance from the sensor to the ground. Due to the growth of the plant, the canopy volume increases and prevents the ultrasonic wave from reaching the ground; as a result, the distance from the sensor to the ground can not be measured. The distance from the sensor to the lower leaves was classified into 3 clusters. Their centers were 43.19, 46.87, and 66.46. All test indexes were very small and proved that the results were reliable. So, we can use the fuzzy ISODATA to deal with the distance data to acquire the locations of the crop canopy and the ground, which are very important for controlling the boom height. However, if the crop was fully covered and no ground was observed, the cluster representing the ground would not be acquired. However, that would not affect the cluster representing the canopy.

The calculation results of fuzzy ISODATA were compared with ones of other methods, such as k-means clustering, mean, and median. The results are listed in Table 2. 

Obviously, the values of fuzzy ISODATA are the closest to the manually measured values. Compared with the k-means clustering algorithm, fuzzy ISODATA has better clustering results. The reason is that the number of clusters is difficult to estimate in k-means clustering. Moreover, the choice of initial cluster center has a greater impact on the clustering results. However, fuzzy ISODATA can dynamically adjust the number and center of clusters to make the clustering results closer to the objective results. As for the methods of computing the mean and median, they all have big errors because they can not exclude the points of lower leaves and ground when calculating the mean and median. However, for fuzzy ISODATA, there are relatively more parameters to be set, and the parameter values are not easy to determine. To get good clustering results, good initial values are required.

### 3.2. Influence of Plant Growth Stage on Calculation Accuracy

**Figure 3:**
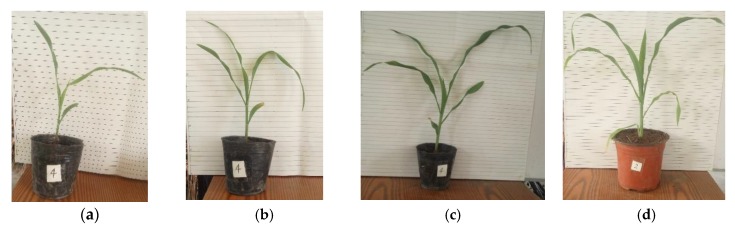
Corn plants at different growth stages: (**a**) 3-leaf stage; (**b**) 4-leaf stage; (**c**) 5-leaf stage; and (**d**) 6-leaf stage.

When the sensor speed was 1 km/h, for corn plants from the 3-leaf stage to the 6-leaf stage (Figure 3), the calculated distances from the sensor to the canopy were compared with the manually measured value. The results are shown in Table 3. 

As shown in Figure 3, at the 3-leaf stage, the plant leaves were short and narrow, so the leaf area was small. Furthermore, the inclination angle of leaves was also small. The ultrasonic echoes bounced from the erect leaves were not detected by the sensor, because these leaves reflected the ultrasonic signal away from the sensor. Consequently, this caused the larger estimation errors of the distance to the canopy at the 3-leaf stage than the ones at other stages. With the growth of the plants, the leaf area increased and the canopy volume became larger. Leaves were gradually extended and tended to be more perpendicular to the sound beam from the sensor. The ultrasonic wave reflected from the canopy increased, and more distance data were able to be used for clustering. Therefore, the calculated error gradually decreased from the 3-leaf stage to the 6-leaf stage. Although the error reached 3.25 cm at the 3-leaf stage, it was not more than 5 cm and met the requirement of boom height control.

### 3.3. Influence of Sensor Moving Speed on Calculation Accuracy

At the 3-leaf stage, the measurement error was larger, owing to the larger inclination angle of leaves. That would make the conclusions less reliable. From the 4-leaf to 6-leaf stage, the leaves became flatter and flatter, and the measurement error was smaller. Moreover, the experiments results showed that the conclusions for the 4-leaf stage are the same with that for the 5-leaf stage and 6-leaf stage. So, we only present the results for the 4-leaf stage.

**Figure 4:**
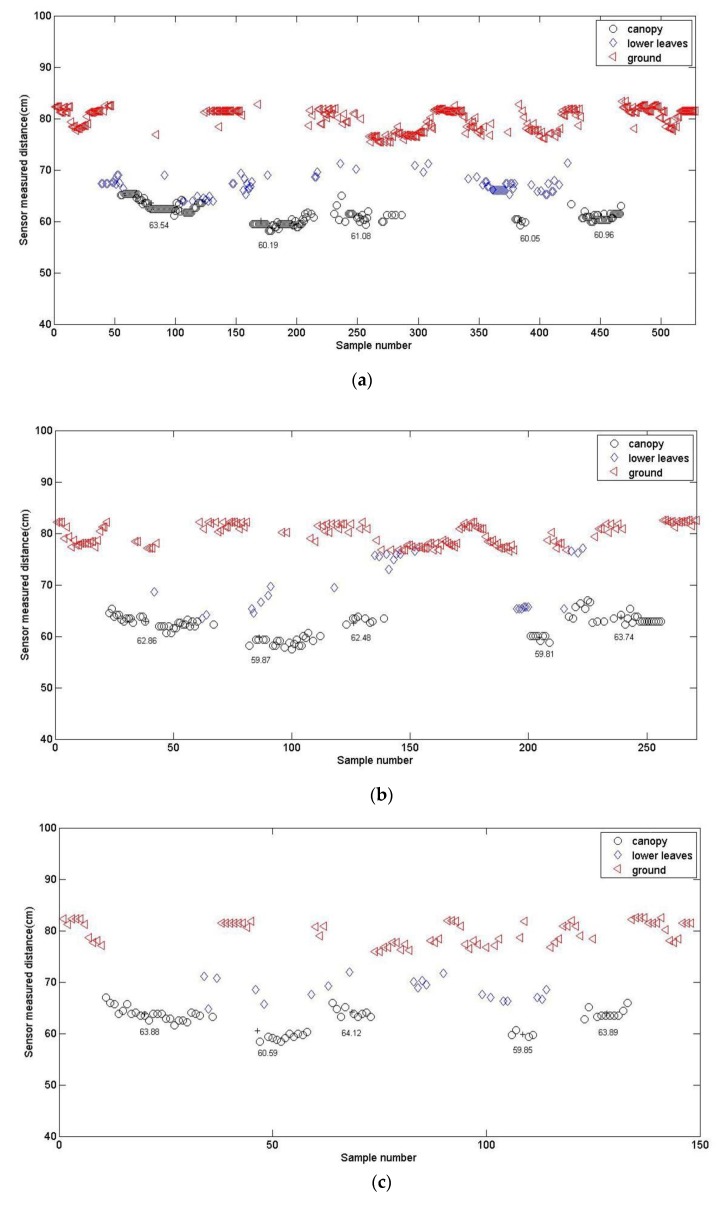

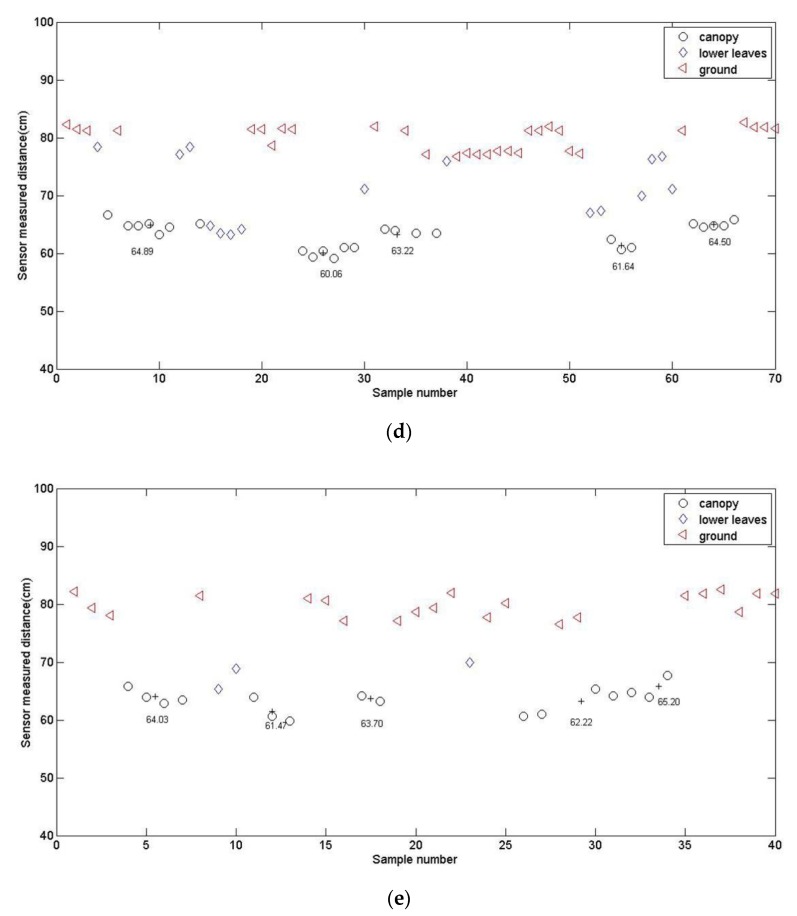
Clustering results for corn plants at the 4-leaf stage at different speeds: (**a**) *v* = 0.5 km/h; (**b**) *v* = 1 km/h; (**c**) *v* = 2 km/h; (**d**) *v* = 4 km/h; (**e**) *v* = 6 km/h.

For five randomly chosen corn plants at the 4-leaf stage, the distances from the sensor to the canopy were calculated at different sensor moving speeds from 0.5 km/h to 6 km/h. The clustering results are demonstrated in Figure 4, and the detailed values are shown in Table 4.

The *F* test result indicated that the variances of calculated values and manually measured values were homogeneous. Therefore, they can be compared by a *t*-test. When the significance level was 0.05, *t*_0.05_ = 2.57, and the *t* values at all speeds were all less than *t*_0.05_. It proved that there were no significantly differences between the calculated values using fuzzy ISODATA and the manually measured values. However, as shown in Table 4, the absolute error increased with the increase of sensor moving speed. The reason was that the faster the sensor moved, the less the amount of data it acquired, which is demonstrated in Figure 4. When the speed reached 6 km/h, there were only about 40 points. Although the collected data were reduced, the distance value from the sensor to the crop canopy could still be obtained and relatively accurate after clustering.

The distribution of the absolute error between the manually measured values and the calculated values was checked using the MATLAB function lillietest(x) at different sensor moving speeds. This function outputs an *h* value and a *p* value. If *h* = 0, the inputed data follows a normal distribution. If *p* > 0.05, the null hypothesis can be accepted. The test result is shown in Table 5. It indicated that the absolute error was normally distributed at the 5% significance level.

Analysis of variance was applied to verify the influence of the sensor moving speed on the absolute error. The results are shown in Table 6. It indicated that the sensor moving speed has a significant influence on the absolute error at the 5% significance level.

### 3.4. Correlation between Calculated Values and Manually Measured Values

**Figure 5:**
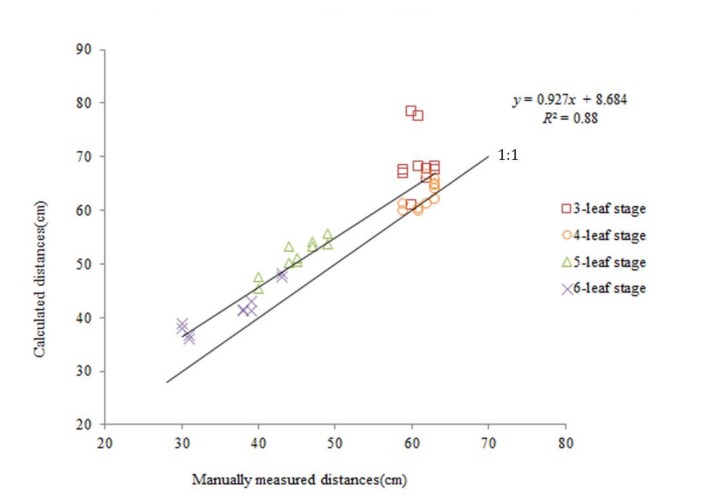
Calculated distances regressed onto manually measured distances from the sensor to the canopy for corn plants including all growth stages.

Regression analysis was used to describe the relationship between calculated values and manually measured values for all growth stages. The calculated distances using fuzzy ISODATA and manually measured distances from the sensor to the canopy were graphed on an XY scatter chart. After the data points were plotted, a trend line, equation, and *R*^2^ were added to illustrate the relationship. The results are shown in Figure 5, which illustrated that the linear regression of calculated distances on manually measured distances had an *R*^2^ of 0.88 with all growth stages included. The results were encouraging, since the calculated distances showed a strong linear correlation with the manually measured distances. The trend for increased correlation with time was similar to the absolute error described before. This trend in corn is intelligible considering the increased size of the plant leaf with advancing growth. Moreover, as the corn plant grew, the increased inclination angle of the leaf made it easier for the canopy to be detected by the sensor, therefore causing the correlation to increase.

The *p*-values of slope and intercept of the regression line were 1.53 × 10^−19^ and 0.00448 separately. They are far less than 0.01, which indicates that the two regression coefficients are highly significant. The residual distribution was tested using the MATLAB function lillietest(x). The result suggested that it did not follow a normal distribution. We found two outliers, the standardized residuals of which are 3.145 and 3.574, respectively. Furthermore, the two data were from the 3-leaf growth stage. Since the leaves were very inclined at this stage, the ultrasonic echo perhaps did not return to the sensor after reflection. That led to the wrong measured data. Consequently, we eliminated the two outliers and performed the regression analysis one more. The new regression equation was *y* = 0.876*x* + 10.587, and the new *R*^2^ value was 0.94. The *p*-values of the two coefficients were 1.38 × 10^−24^ and 1.43 × 10^−6^ separately. It indicates that they are more significant than those of the old regression equation. In addition, the residual was normally distributed after elimination of the two outliers. The standard errors of regression coefficients were calculated for different growth stages. They were 0.56, 0.39, 0.14, 0.10 for the slope and 34.06, 23.96, 6.45, 3.79 for the intercept respectively from the 3-leaf to the 6-leaf growth stage. The smaller the error, the more accurate the coefficient. The errors decreased with the plant growth. In addition, we calculated the leverage of all points. The results demonstrated that there was not a high leverage point. None of points can strongly influence the slope of the regression line.

## 4. Conclusions

In this paper, ultrasonic ranging was combined with fuzzy ISODATA to obtain the distances from the sensor to the crop canopy for corn plants from the 3-leaf stage to the 6-leaf stage. The calculated values had a high correlation with the manually measured values. Applying the proposed method, we can acquire the distances from the sensor to the canopy and the ground at the same time for plants from the 3-leaf stage to the 5-leaf stage. After the 6-leaf stage, the distances to the ground cannot be obtained due to the increased blockage of sound waves by plant leaves. With the growth of plants, the error of calculated values decreased. The increased sensor moving speeds led to increased error. However, the errors were all less than 5 cm and acceptable in field application. 

Our development can be used in the boom height control system of sprayers. We can install one ultrasonic sensor on each end of the boom to measure the distance from the boom to the crop canopy or the ground. The data were processed with a computer to judge if the distance was appropriate. If not, the computer would send a control signal to a hydraulic cylinder or an electrical motor to lift or lower either wing section of the boom. So, the boom can be maintained at a constant or near constant distance.

However, there are still some limitations in our work. The sampling rate of ultrasonic sensors is lower. So, the acquired data are fewer when the sensor moves at a higher speed. That will affect the clustering accuracy. The determination of parameters of ISODATA is difficult and requires full understanding of them and sufficient experience. Some outdoor conditions, such as the air temperature, humidity, and wind velocity, can influence the performance of ultrasonic sensors. When the sprayer works, uneven soil surface causes the vibration of boom, which may affect the distance measurement. Moreover, the mutual occlusion of leaves of different crops may result in more extraordinary data.

In the future, more research is needed to learn how the beam angle of ultrasonic sensors affects the measurement errors and calculation results. Some filter algorithms should be developed to eliminate the vibration effect and the abnormal data before clustering. The research subject should be extended to other crops, such as wheat, cotton, peanut, and so on. In addition to lab experiments, field experiments should also be conducted to verify the adaptability of our work. In order to achieve more data, laser sensors can be applied to take the place of ultrasonic sensors.

## Figures and Tables

**Table 1:** Cluster center and test index.

Growth Stage	Number of Clusters	Cluster Center Value (cm)			
3-leaf stage	2	80.17	63.25		
4-leaf stage	3	79.22	65.53	59.81	
5-leaf stage	4	80.90	66.14	51.21	44.26
6-leaf stage	4	66.46	46.87	43.19	39.71

**Table 2:** Calculated and manually measured values of distance from the sensor to the canopy with different algorithms. ISODATA: Iterative Self-Organizing Data Analysis Technique Algorithm.

Growth Stage	Manually Measured Value (cm)	Fuzzy ISODATA (cm)	K-Means Clustering (cm)	Mean (cm)	Median (cm)
3-leaf stage	60	63.25	63.65	74.22	78.6
4-leaf stage	57	59.81	65.52	74.33	77.70
5-leaf stage	46	44.26	43.89	55.05	50.75
6-leaf stage	38	39.71	42.00	45.18	43.1

**Table 3:** Calculated and manually measured values of distance from the sensor to the canopy at different growth stages.

Growth Stage	Calculated Value (cm)	Manually Measured Value (cm)	Absolute Error (cm)
3-leaf stage	63.25	60	3.25
4-leaf stage	59.81	57	2.81
5-leaf stage	44.26	46	1.74
6-leaf stage	39.71	38	1.71

**Table 4:** Calculated and manually measured values of distance from the sensor to the canopy at different sensor moving speeds.

Plant Number	Manually Measured Value (cm)	Calculated Value at Different Speed (cm)				
		0.5 km/h	1 km/h	2 km/h	4 km/h	6 km/h
Plant no. 1	61	63.54	62.86	63.88	64.89	64.03
Plant no. 2	59	60.19	59.87	60.59	60.06	61.47
Plant no. 3	60	61.08	62.48	64.12	63.22	63.7
Plant no. 4	57	60.05	59.81	59.85	61.64	62.22
Plant no. 5	61	60.96	63.74	63.89	64.5	65.2
Mean values	59.6	61.164	61.752	62.466	62.862	63.324
Absolute error		1.564	2.152	2.866	3.262	3.724
*t* value		0.15	0.09	0.04	0.02	0.01

**Table 5:** Normal distribution test result of absolute error.

Value	*V* = 0.5 km/h	*V* = 1 km/h	*V* = 2 km/h	*V* = 4 km/h	*V* = 6 km/h
*h*	0	0	0	0	0
*p*	0.5	0.3481	0.1853	0.2078	0.5

Note: when *p* is greater than the largest tabulate value in MATLAB, it is set 0.5.

**Table 6:** Variance analysis result of absolute error.

Source	Sum of Squares of Deviation	Degree of Freedom	Mean Squares of Deviation	*F* Value	Significance
Groups	14.91	4	3.73	3.16	*
Error	23.64	20	1.18		
Total	38.55	24			

Note: *F*_0.05_ (4, 20) = 2.86, “*” denotes significance.
